# Community-based Health Research: Issues and Methods

**DOI:** 10.3201/eid1102.041033

**Published:** 2005-02

**Authors:** William W. Darrow

**Affiliations:** * Florida International University, North Miami, Florida, USA

**Keywords:** health research, book review

So many, 11 of 14, contributors to this volume are from the Atlanta area that I expected this book to speak with a Southern drawl. That it does not attests to how much this metropolis, growing in kudzu-fashion, has changed. This element of profound change is also a major motif in the 10 chapters collected by the editors to promote community-based health research as a mechanism for addressing historic wrongs.

The book is aptly titled and subtitled. Former Surgeon General David Satcher writes in a concise but illuminating foreword, "Community-based research is where medicine, public health, and science meet." In the opening chapter, Daniel Blumenthal and Elleen Yancey herald the arrival of a "new paradigm" in which community members become full partners with "culturally competent" researchers. To them, community-based research is population centered, prevention focused, multidisciplinary, collaborative, enlightening, and empowering. Caswell Evans follows by adding "evidence-based" assessments, findings, and guidance to the mix. In chapter 3, Bill Jenkins, Camara Jones, and Blumenthal address some of the ethical issues related to community-based research by describing, analyzing, and drawing lessons from the Tuskegee syphilis study. Culturally and linguistically diverse voices from the community are heard in chapter 4.

Attention shifts from issues to methods in the last 6 chapters of the book. In textbook fashion, Nabih R. Asal and Laura A. Beebe distinguish observational studies from experimental designs in chapter 5 and remind the reader of the importance of person, place, and time in epidemiologic investigations. The strengths and weaknesses of the Behavioral Risk Factor Surveillance System are illustrated by Deborah Holtzman in chapter 6. Qualitative research methods are described in chapter 7 and applied to a case study of 45 African-American, female crack-cocaine users in chapter 8. Community intervention trials are introduced and a half dozen are reviewed in chapter 9. Then the book rather abruptly ends with a short chapter on cardiovascular risk-reduction community intervention trials.

Instructors looking for a graduate-level textbook may find this recent addition to the preventive medicine literature incomplete. It fails to link community-based research with theories of social and cultural change; the principles and practices of community mobilization; and the identification, development, implementation, and evaluation of culturally competent interventions. The editors have produced an adequate introduction to community-based research issues and methods, but a concluding section that serves to pull all the components together would put additional copies of this publication in college bookstores.

